# Contactin-associated protein 2 autoantibodies can be associated with multifocal motor-like neuropathy: a case report

**DOI:** 10.1177/17562864231189323

**Published:** 2023-08-16

**Authors:** Louisa Müller-Miny, Raoul Sauer, Andreas Schulte-Mecklenbeck, Catharina C. Gross, Stjepana Kovac, Matthias Schilling, Carolin Beuker, Heinz Wiendl, Gerd Meyer zu Hörste

**Affiliations:** Department of Neurology with Institute of Translational Neurology, University Hospital Münster, Münster, Germany; Department of Neurology with Institute of Translational Neurology, University Hospital Münster, Münster, Germany; Department of Neurology with Institute of Translational Neurology, University Hospital Münster, Münster, Germany; Department of Neurology with Institute of Translational Neurology, University Hospital Münster, Münster, Germany; Department of Neurology with Institute of Translational Neurology, University Hospital Münster, Münster, Germany; Department of Neurology with Institute of Translational Neurology, University Hospital Münster, Münster, Germany; Department of Neurology with Institute of Translational Neurology, University Hospital Münster, Münster, Germany; Department of Neurology with Institute of Translational Neurology, University Hospital Münster, Münster, Germany; Department of Neurology with Institute of Translational Neurology, University of Münster, Albert-Schweitzer-Campus 1, Building A1, Münster 48149, Germany

**Keywords:** case report, CASPR2, flow cytometry, multifocal motor neuropathy

## Abstract

Autoantibodies against contactin-associated protein 2 (CASPR2) are usually associated with autoimmune encephalitis and neuromyotonia. Their association with inflammatory neuropathies has been described in case reports albeit all with distal symmetric manifestation. Here, we report a patient who developed distal arm paresis, dominantly of the right arm, over the course of 1 year. Electroneurography showed a conduction block of motor nerve conduction, nerve ultrasonography a swelling of the right median and ulnar nerve and flow cytometry an increase in natural killer (NK cells) in the blood and natural killer T (NKT) cells in the cerebrospinal fluid (CSF), therefore indicating a multifocal motor neuropathy-like (MMN-like) phenotype. CASPR2 autoantibodies were detected in serum and CSF. Through immunotherapy with intravenous immunoglobulins the patient showed clinical and neurographic improvement. We therefore describe the first association of CASPR2 autoantibodies with a MMN-like clinical manifestation, extending the spectrum of CASPR2-associated diseases.

## Introduction

Autoantibodies against contactin-associated protein 2 (CASPR2) are directed against a component of the voltage-gated potassium channel complex and have commonly been associated with autoimmune encephalitis and neuromyotonia.^1^ Clinical manifestations range from peripheral nerve hyperexcitability and chronic pain to seizures.^2,3^ CASPR2 antibodies have been shown to interrupt the positioning at the juxtaparanodal region in the nodes of Ranvier.^4^ However, mechanisms behind the large range of CASPR2-associated diseases remain only partially characterized. Further disease entities associated with CASPR2 antibodies have been described.^5,6^ Reports associating CASPR2 autoantibodies with peripheral neuropathies are rare and have so far only described a distal symmetric distribution of peripheral neuropathy.^5,7^ Here, we are the first to describe an association of CASPR2 autoantibodies with multifocal motor neuropathy-like (MMN-like) clinical manifestation with response to immunotherapy with intravenous immunoglobulins (IVIGs).

## Case report

### Patient information

A 49-year-old male presented to our clinic for further diagnostic evaluation of right arm paresis. The patient reported an increasing weakness of the right hand in the preceding months. He could no longer carry objects with his outstretched right arm and had an increasing difficulty in his fine motor skills such as buttoning shirts. The patient initially noticed dysaesthesia of his fingertips in his right hand 2 years ago, which had not bothered him significantly and did not, in contrast to his motor deficits, progress over time. Magnetic resonance imaging (MRI) of the head and cervical spine showed no abnormalities while initial treatments with physiotherapy and oral cortisone had been performed, without any improvement.

The clinical neurological examination on admission revealed a paresis dominantly of the right distal arm but no atrophy. Specifically, he displayed motor deficits (Medical Research Council grading system) in his wrist flexion [right (r): 4−/5, left (l): 5/5], wrist extension (r: 4/5, l: 5/5), wrist abduction [ulnar: (r: 4/5, l: 5/5), radial: (r: 4+/5, l: 5/5)], finger extension and flexion (r: 4+/5, l: 5/5) in addition to his finger spread (r: 4/5, l: 5/5). His thumb abduction, opposition (r: 4/5, l: 5/5), and extension (r: 4−/5, l: 5/5) showed deficits on the right side. There was a paresis in the external shoulder rotation on both sides (l, r: 4+/5) but no deficits in any other muscles (l/r: 5/5). The patient also presented an isolated hypoesthesia in the fingertips (one third of distal phalanx) of his first three fingers on both sides with an exclusion of the thumb pad and palm without thermdysesthesia and pallhypesthesia. Cramps and fasciculations were not observed. Reflexes were weak but equal on both sides. The neurological exam was otherwise unremarkable.

### Diagnostic assessment

**Figure 1.**
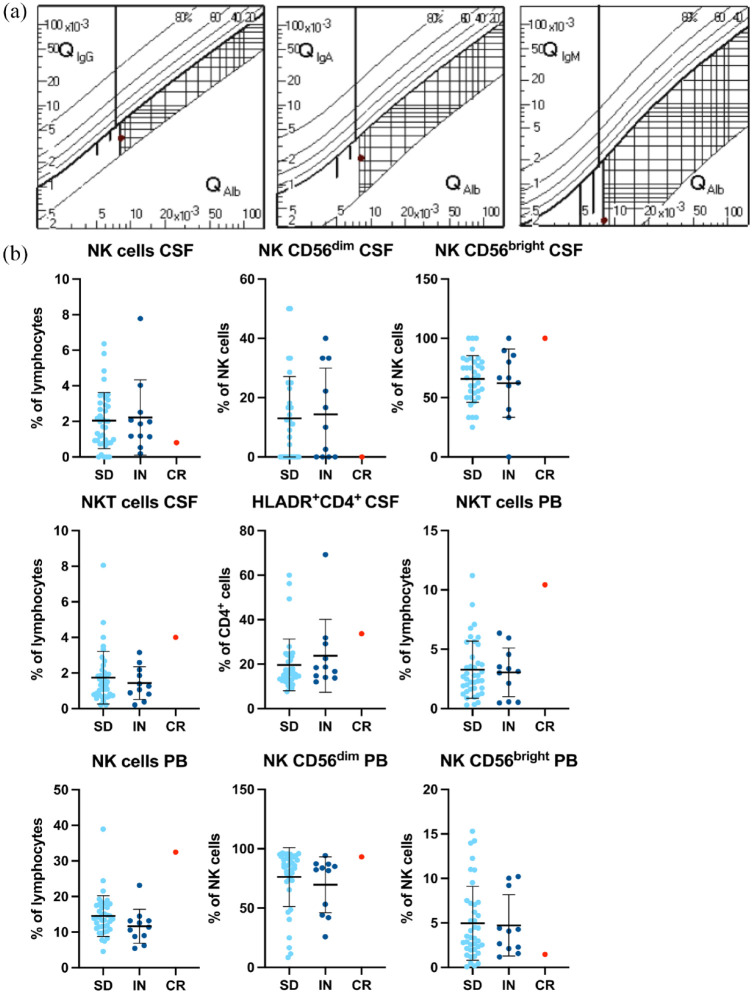
Patients flow cytometry supports MMN-like diagnosis. Projection of the immunoglobulin (Ig) CSF/Ig serum index compared to the albumin CSF/albumin serum index at the time of sample collection. (a) Reiber scheme for IgG, IgM, and IgA. The red dots indicate the calculated quotient. The albumin quotient indicates a blood-CSF barrier dysfunction. (b) Selected flow cytometry parameters in percent (Supplemental Figure 2 for gating scheme) of peripheral blood and CSF compared to patients with inflammatory neuropathy and non-inflammatory control patients. Red dot indicates the anti-CASPR2 + MMN-like patients (CR) values. Light blue dots depict values of 39 age-matched non-inflammatory controls [i.e. somatoform disorder patients (SD)with no signs indicating an inflammatory CSF]. Dark blue dots depict 11 age-matched inflammatory neuropathy patients [7x chronic inflammatory demyelinating polyneuropathy (CIDP), 2x multifocal motor neuropathy (MMN), and 2x Guillain–Barre syndrome (GBS)] for comparison. Line and error bars depict mean + standard deviation. CASPR2, contactin-associated protein 2; CSF, cerebrospinal fluid; MMN-like, motor neuropathy-like.

A diagnostic lumbar puncture and subsequent cerebrospinal fluid (CSF) analysis showed a normal cell count and slightly increased total protein levels (603 mg/l) with a blood-CSF barrier dysfunction as quantified by the albumin quotient, but no intrathecal immunoglobulin synthesis (Figure 1). Flow cytometry analysis revealed an increase of activated HLA-DR expressing CD4^+^ T cells (33.7%) in the CSF compared to age-matched non-inflammatory controls [Figure 1(b)]. Furthermore, increased proportions of natural killer cells (NK cells) with a CD56^bright^ to CD56^dim^ shift were observed in the blood of the patient compared to both non-inflammatory controls and other inflammatory neuropathy patients. Both blood and CSF displayed higher levels of NKT cells compared to the non-inflammatory controls. Interestingly, we detected antibodies against CASPR2 in the CSF (1:32) and in the serum of the patient (1:320) (Table 1) (indirect immunofluorescence assay). Serum ganglioside antibody determination showed negative Immunoglobulin (Ig)G antibodies and a borderline positive result for IgM antibodies against GM3 and GQ1b in the serum, which, based on the assay guidelines, was considered negative (Table 1) (enzyme immunoassay).

**Table 1.** Basic CSF parameters and antibody concentrations.

CSF parameters	Patient CSF
Cells/µl	0
Lymphocytes/µl	0
Granulocytes/µl	0
Erythrocytes/µl	0
Total protein (mg/l)	603
Albumin CSF (mg/l)	340
Albumin serum (g/l)	41.6
Albumin ratio	8.2
IgG CSF (mg/l)	31.7
IgG serum (g/l)	7.9
IgG ratio	4
IgA CSF (mg/l)	2.35
IgA serum (g/l)	1.08
IgA ratio	2.2
IgM CSF (mg/l)	0.38
IgM serum (g/l)	1.06
IgM ratio	0.4
Oligoclonal Bands type	1
Glucose ratio	0.89
Lactate (mmol/l)	1.79
CASPR2 CSF	1:32
CASPR2 serum	1:320
Ganglioside profile serum (IgG) (anti-GM1, anti-GM2, anti-GM3, anti-GD1a, anti-GD1b, anti-GT1b, anti-GQ1b)	Negative
Ganglioside profile serum (IgM) (anti-GM1, anti-GM2, anti-GD1a, anti-GD1b, anti-GT1b)	Negative
Anti-GM3 IgM serum	Borderline positive
Anti-GQ1b IgM serum	Borderline positive

CASPR2, contactin-associated protein 2; CSF, cerebrospinal fluid.

**Figure 2.**
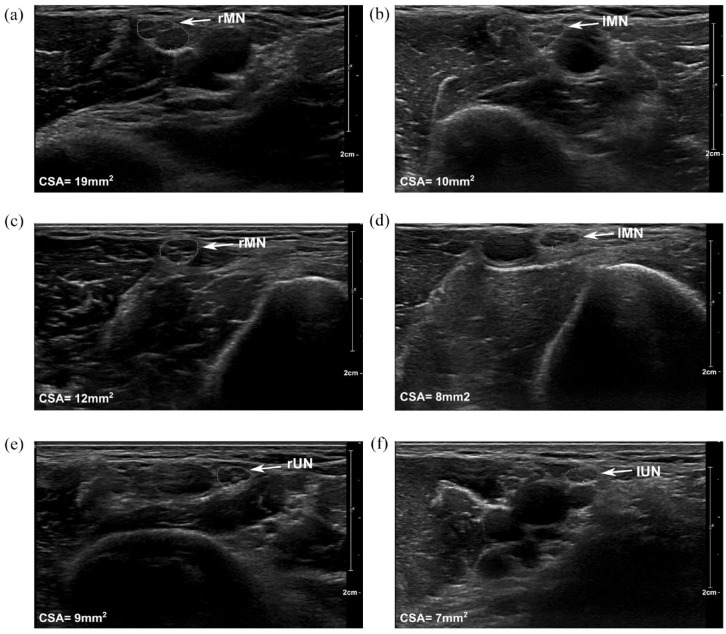
Ultrasonography shows enlargement of peripheral nerves. Ultrasonography of peripheral nerves in the right (a, c, e) and left (b, d, f) arm. Enlargement of right median nerve (rMN) in the proximal (a) and middle (c) upper arm compared to the left median nerve (lMN) (b, d). Enlargement of fascicles of the right (e) ulnar nerve (rUN) in the proximal upper arm compared to the left ulnar nerve (lUN) (f). Ultrasound depth depicted as a scale on the right, cross-sectional area (CSA) of the respective nerve on the bottom left. Circled area depicts the measured nerve.

Further diagnostics showed a motor nerve conduction block at Erb’s point-axilla with area reduction of >50% (Table 2) in the right ulnar nerve. Nerve conduction velocity showed normal values in the median and ulnar nerve. F-wave latencies of the ulnar nerves were prolonged and the right median nerve was not measurable. The radial and tibial nerve on both sides showed normal functions. The sensory nerve conduction was normal in all of the above-mentioned nerves suggestive of an isolated motoric axonal and demyelinating neuropathy. The needle electromyography showed chronic neurogenic changes in the abductor pollicis brevis muscle, with no abnormal activity at rest. We diagnosed a MMN as the patient fulfilled the diagnostic criteria.^8^ A peripheral nerve ultrasonography (Figure 2) showed an enlargement of the cross-sectional areas^9,10^ of the right median nerve and discontinuous swelling of individual fascicles of the right median and ulnar nerve in the upper arm supporting the MMN-like phenotype.

**Table 2.** Nerve conduction study parameters.

Motor	Before treatment							After IVIG treatment						
	Latency (ms)	NCV (m/s)	Amplitude (mV)	Amplitude-diff (%)	Area-diff (%)	Stim-intensity (mA)	Min. F-latency (ms)	Latency (ms)	NCV (m/s)	Amplitude (mV/µV)	Amplitude -diff (%)	Area-diff (%)	Stim-intensity (mA)	Min. F-latency (ms)
Median nerve right														
Wrist-APB	4.40		2.4			18.1	nr	4.22		7.6			100	18.2
Elbow-wrist/APB	9.85	48.6	2.5	4.2	−5.6	14.3		9.73	54.4	1.71	−77.5	−76.6	100	
Axilla-elbow/APB	12.9	41.0	2.4	−4.0	−19.8	48.2		12.6	52.3	4.4.	157	90.2	61.0	
Erb-axilla/APB	17.5	71.7	2.0	−16.7	−25.9	89.8		19.4	41.2	3.4	−22.7	−33.3	100	
Median nerve left														
Wrist-APB	3.98		3.2			12.3	29.0	4.5		9.5			100	28.5
Elbow-wrist/APB	9.04	52.4	3.1	−3.1	−5.6	25.0		8.98	55.8	2.0	−78.9	−75.0	100	
Axilla-elbow/APB	11.8	52.5	3.2	3.2	1.98	13.6		11.1	70.8	7.8	290	193	21.8	
Erb-axilla/APB	14.2	95.8	3.8	18.8	53.4	37.8		15.1	67.5	6.9	−11.5	−7.5	100	
Ulnar nerve right														
Wrist-ADM/ADM	2.81		8.0			22.4	36.4	3.23		9.3			17.1	25.4
Lower elbow-wrist/ADM	7.25	50.7	5.5	−31.3	−12.3	78.2		7.9	49.3	7.0	−24.7	−3.3	80.6	
Upper elbow–lower elbow/ADM	8.79	51.9	5.7	3.6	3.1	27.4		9.54	42.7	6.9	−1.43	−1.15	49.4	
Axilla-upper elbow/ADM	11.6	46.3	4.3	−24.6	−25.5	54.0		11.7	50.9	6.1	−11.6	−0.78	49.4	
Erb-axilla/ADM	16.9	58.5	0.71	−83.5	−81.1	69.8		18.4	41.8	1.92	−68.5	−67.2	100	
Ulnar nerve left														
Wrist-ADM/ADM	2.58		6.4			59.0	33.1	2.77		7.8			60.6	29.8
Lower elbow-wrist/ADM	7.31	48.6	5.5	−15.4	−15.0	56.2		7.19	52.0	6.3	−19.2	−5.5	87.6	
Upper elbow-lower elbow/ADM	8.56	48.0	5.6	1.82	2.2	76.0		8.27	64.8	7.0	11.1	8.7	87.6	
Axilla-upper elbow/ADM	11.1	51.2	5.1	−8.9	7.0	29.0		11.0	47.6	6.7	−4.3	−8.8	6.7	
Erb-axilla/ADM	14.8	66.2	5.0	−1.96	−4.1	71.6		15.5	51.1	6.0	−10.4	−4.6	64.6	
Tibial nerve right														
Ankle-Abd. hal/Abd. hal.	5.38		5.8			100	58.7							
Knee-ankle/Abd. hal.	15.3	41.8	4.0	−31.0	8.3	86.2								
Tibial nerve left														
Ankle-Abd. hal./Abd hal.	3.90		5.8			89.8	61.1							
Knee-ankle/Abd hal.	14.4	41.0	4.1	−28.1	−4.3	100								
Radial nerve right														
Lower arm-EDC/EDC	4.13		5.4			100								
Upper elbow-lower arm/EDC	6.06	82.9	6.3	16.7	16.7	73.4								
Radial nerve left														
Lower arm-EDC/EDC	3.98		6.0			100								
Upper elbow-lower arm/EDC	6.54	66.4	6.2	3.3	11.0	72.4								
Sensory	Latency (ms)	Amplitude (µV)	NCV (m/s)					Latency (ms)	Amplitude (µV)	NCV (m/s)				
Median nerve right (wrist-dig. II)	2.45	10.2	65.3					2.46	6.1	69.1				
Median nerve left (wrist-dig. II)	2.69	15.5	57.6					3.00	13.4	56.7				
Ulnar nerve right (wrist-dig. V)	2.18	11.2	61.9					2.27	7.0	57.3				
Ulnar nerve left (wrist-dig. V)	1.99	16.1	67.8					2.21	16.6	63.3				

Abd. hal, abductor hallucis muscle; ADM, abductor digiti minimi muscle; APB, abductor pollicis brevis muscle; diff, difference; dig, digitus manus; EDC, extensor digitorum communis; IVIG, intravenous immunoglobulin; m/s, meter/second; mA, milliampere; ms, milliseconds; NCV, nerve conduction velocity; nr, not recordable; Stim-intensity, stimulation intensity; µV, microvolt.

A neuropsychiatric consultation showed a normal neurocognitive performance profile with no indication of an autoimmune encephalitis profile. In context with the clinical presentation and his normal brain MRI we thus ruled out an autoimmune encephalitis. Due to the CASPR2 antibodies, which can be associated with malignancy,^3^ we initiated a tumor search with an abdomen and thyroid sonography as well as an urologic consultation and Positron emission tomography–computed tomography (PET-CT) which showed no indication of a tumor.

### Follow-up and outcome

In a follow-up visit the patient showed signs of additional deficits on the left side. Specifically, he had motor deficits in his right finger extension (r: 4/5, l: 4+/5), his thumb abduction (r: 4/5, l: 4+/5), as well as thumb extension (r: 4−/5, l: 4/5). We started treatment with IVIG every 4 weeks. Three months later he reported a clinical improvement in his daily activities as he was now able to open drawers again. Objective parameters included an improvement of his finger extension (r: 4/5; l: 5/5) and his wrist extension (r: 5/5; l: 4+/5) and grip strength (Martin-Vigorimeter) (l: 0.6 bar; r: not measurable; 3-month follow-up = l: 0.86 bar, r: 0.36 bar). We also measured an improved electroneurography, with improved conduction velocity and amplitude as well as a decreased area reduction in the conduction block (Table 2) but a new motor nerve conduction block with an area reduction of >50% on the median nerve at the elbow on both sides. In the follow-up, CASPR2 antibodies were confirmed in the serum (1:10) with an indirect immunofluorescence assay but not measured in the CSF, therefore also presenting a treatment response.

### Differential diagnosis

Further differential diagnosis included Lewis-Sumner syndrome and amyotrophic lateral sclerosis as well as other more common causes of CASPR2 antibodies.

We could not find any signs of the first motor neuron in the clinical examination. While the patient described sensory symptoms in the innervation area of the median nerve bilaterally, they did not extend to the rest of its innervation area (thumb pad, lower arm) and did not progress over time. In addition, we could not find deficits in other sensory qualities or the sensory nerve conduction to objectify these deficits, indicating a Lewis-Sumner syndrome.

However, we do acknowledge that the inflammatory neuropathies are most likely an overlapping disease spectrum, which is why we also call it MMN-like disease. Further diagnostics in our case also showed no indication of more common diseases associated with CASPR2 antibodies.

## Discussion

Here we report a case of multifocal motor-like neuropathy with corresponding electrophysiological changes that is unusual in its association with CASPR2 antibodies. We thus describe for the first time that anti-CASPR2 antibodies can be associated with MMN-like disease.

The MMN-like diagnosis was clinically based on upper-limb weakness with an initial onset of the median and radial nerve of the right hand with an additional affection of the left radial and median nerve in the follow-up. Further objectifiable parameters included a nerve conduction block in the NCS and an enlargement of the median nerve in the ultrasonography. The CSF showed an increase of the CSF protein in accordance with inflammatory neuropathy (Heming 2020)^11^ and the patient had a treatment response to IVIG therapy.

Anti-CASPR2 antibodies are usually associated with autoimmune encephalitis and neuromyotonia. Few case reports describe an association with peripheral immune neuropathy. Examples include singular patients with peripheral motor neuron hyperexcitability and optic neuritis, autonomic symptoms and polyneuropathy,^5^ or chronic inflammatory demyelinating neuropathy.^7^ An association of CASPR2 with MMN-like disease is biologically plausible as the targeted glycoprotein of anti-CASPR2 is located directly at the nodes of Ranvier and, similarly to targets of other antibodies associated with an inflammatory neuropathy,^12–16^ play a role in the saltatory potential transduction.

Our flow cytometry data (Figure 1, Supplemental Figure 1) further underline our MMN-like diagnosis by showing an increase of NK cells previously shown in a murine model of Guillain–Barre syndrome at the affected nerve.^17^ The treatment response through IVIGs supports the diagnosis and shows tentative evidence of treatment response for CASPR2-associated neuropathies that is unusual for IgG4-mediated diseases where treatment response of other IgG4-mediated neuropathies has been shown in only 10–20%.^16,18^ B cell depletion therapy could represent an alternative therapeutic approach. The predominantly peripheral immune changes associated with CASPR2 antibodies in context with further cases of autoimmune neuropathies indicate a larger role of the peripheral immune system in anti-CASPR2-associated diseases.

In summary, we, for the first time, present a case of MMN-like disease associated with anti-CASPR2 autoantibodies. This supports the hypothesis that anti-CASPR2 antibodies can also trigger motor-dominant peripheral immune neuropathies.

## Supplemental Material

sj-png-1-tan-10.1177_17562864231189323 – Supplemental material for Contactin-associated protein 2 autoantibodies can be associated with multifocal motor-like neuropathy: a case report Click here for additional data file. Supplemental material, sj-png-1-tan-10.1177_17562864231189323 for Contactin-associated protein 2 autoantibodies can be associated with multifocal motor-like neuropathy: a case report by Louisa Müller-Miny, Raoul Sauer, Andreas Schulte-Mecklenbeck, Catharina C. Gross, Stjepana Kovac, Matthias Schilling, Carolin Beuker, Heinz Wiendl and Gerd Meyer zu Hörste in Therapeutic Advances in Neurological Disorders

sj-png-2-tan-10.1177_17562864231189323 – Supplemental material for Contactin-associated protein 2 autoantibodies can be associated with multifocal motor-like neuropathy: a case report Click here for additional data file. Supplemental material, sj-png-2-tan-10.1177_17562864231189323 for Contactin-associated protein 2 autoantibodies can be associated with multifocal motor-like neuropathy: a case report by Louisa Müller-Miny, Raoul Sauer, Andreas Schulte-Mecklenbeck, Catharina C. Gross, Stjepana Kovac, Matthias Schilling, Carolin Beuker, Heinz Wiendl and Gerd Meyer zu Hörste in Therapeutic Advances in Neurological Disorders

## Declarations

**Ethics approval and consent to participate:** Not applicable for case reports.

**Consent for publication:** The patient has provided a written informed consent to publication of this article.

**Availability of data and materials:** Anonymized data can be made available upon reasonable request.
