# Supplementary figures and images for: Contactin-associated protein 2 autoantibodies can be associated with multifocal motor-like neuropathy: a case report
Source: Ther Adv Neurol Disord. 2023 Aug 16;16:17562864231189323. doi: 10.1177/17562864231189323 (PMC10434843; doi:10.1177/17562864231189323)

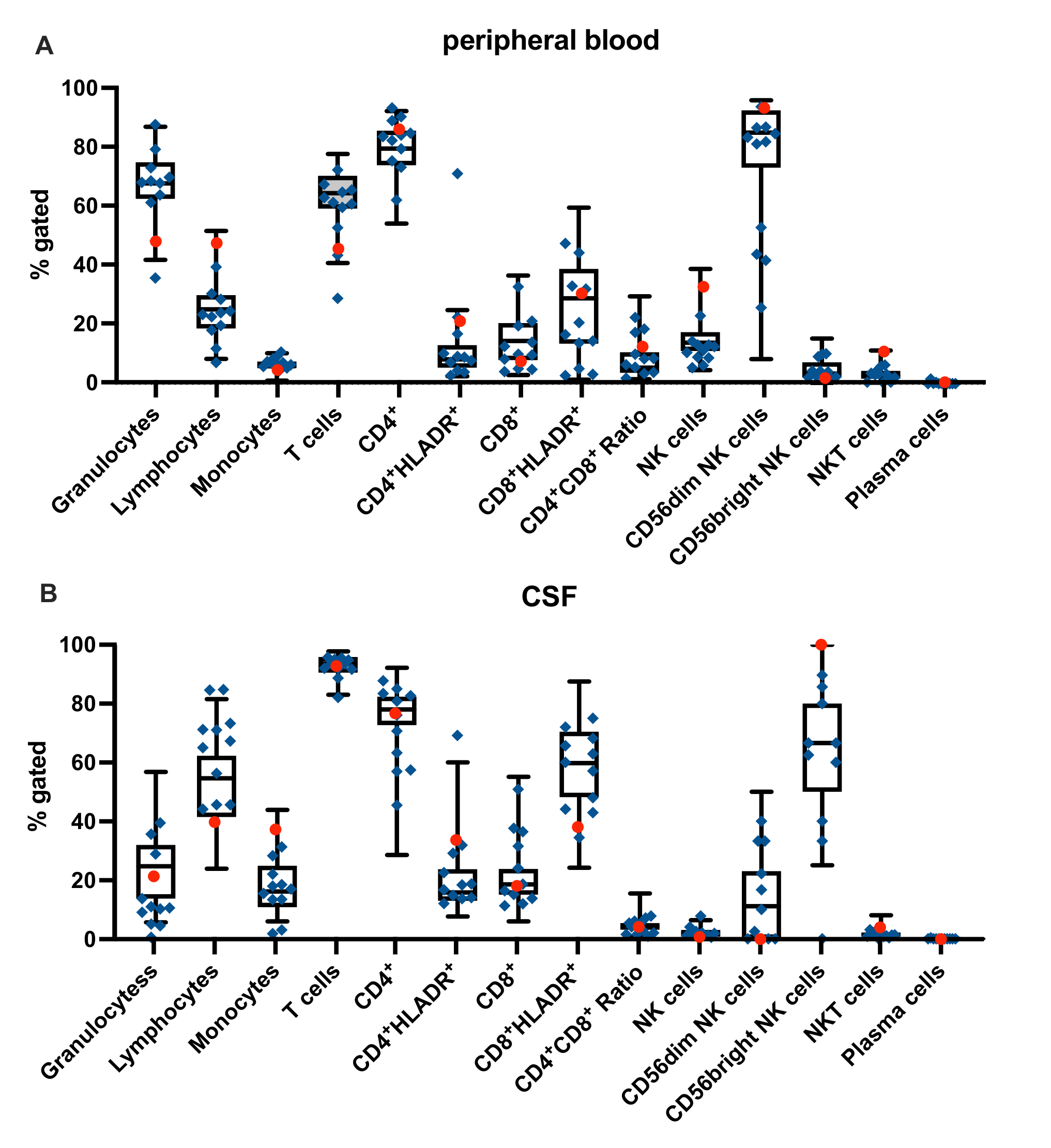

Supplement: sj-png-1-tan-10.1177_17562864231189323 – Supplemental material for Contactin-associated protein 2 autoantibodies can be associated with multifocal motor-like neuropathy: a case report [file sj-png-1-tan-10.1177_17562864231189323.png]

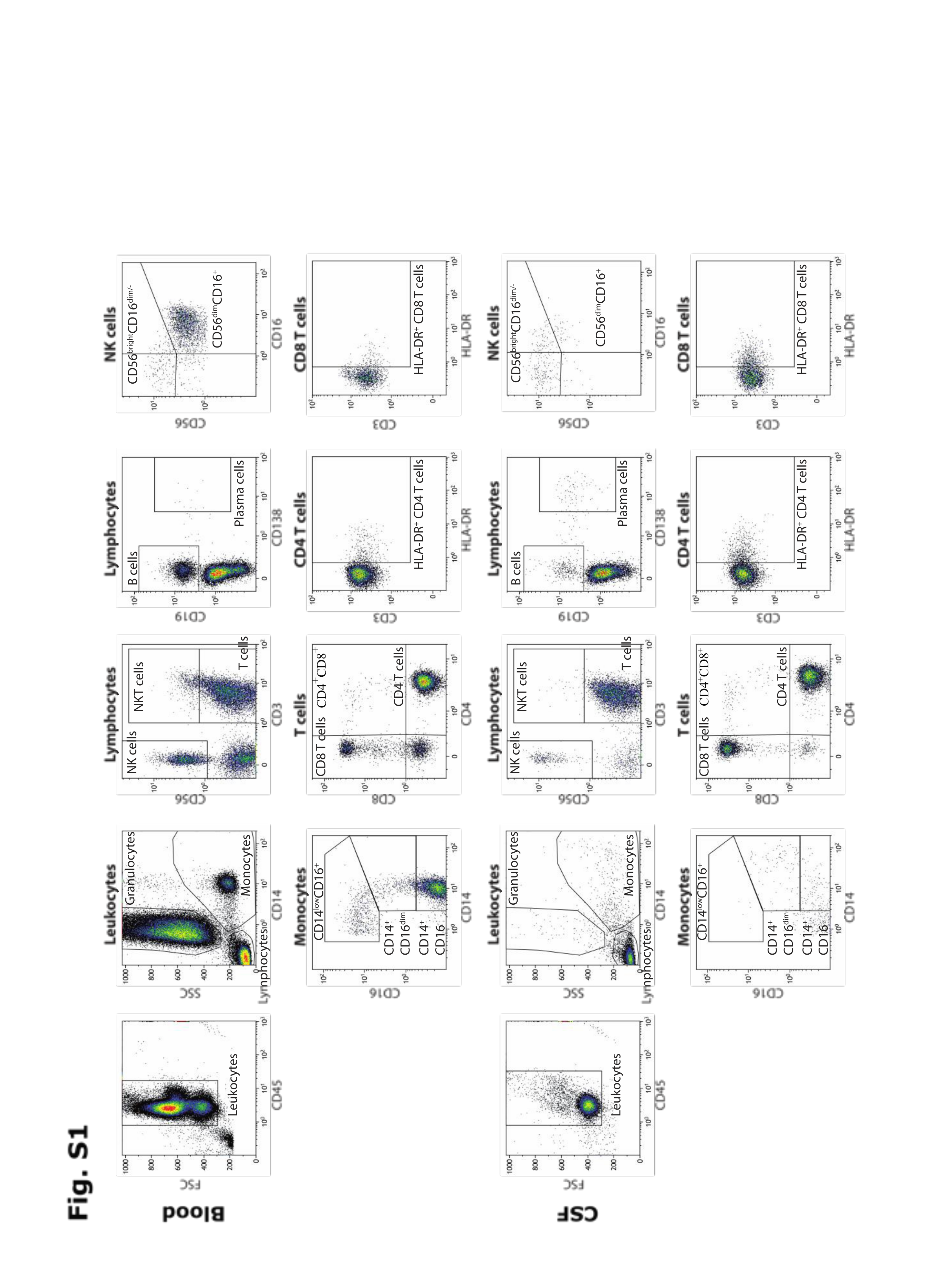

Supplement: sj-png-2-tan-10.1177_17562864231189323 – Supplemental material for Contactin-associated protein 2 autoantibodies can be associated with multifocal motor-like neuropathy: a case report [file sj-png-2-tan-10.1177_17562864231189323.png]
